# 4-{2-[2-(4-Formyl­phen­oxy)eth­oxy]eth­oxy}benzaldehyde

**DOI:** 10.1107/S1600536811018150

**Published:** 2011-05-25

**Authors:** Zhen Ma, Yiqun Cao

**Affiliations:** a School of Chemistry and Chemical Engeneering, Guangxi University, Guangxi 530004, People’s Republic of China

## Abstract

The title compound, C_18_H_18_O_5_, was obtained by the reaction of 4-hy­droxy­benzaldehyde with bis­(2,2-dichloro­eth­yl) ether in dimethyl­formamide. In the crystal, the mol­ecule lies on a twofold rotation axis that passes through the central O atom of the aliphatic chain, thus leading to one half-mol­ecule being present per asymmetric unit. The carbonyl, aryl and O—CH_2_—CH_2_ groups are almost coplanar, with an r.m.s. deviation of 0.030 Å. The aromatic rings are approximately perpendicular to each other, forming a dihedral angle of 78.31 sh;H⋯O hydrogen bonds and C—H⋯π inter­actions help to consolidate the three-dimensional network.

## Related literature

For the synthesis and structures of dialdehydes, see Aravindan *et al.* (2003 ▶); Han & Zhen (2005 ▶); Ma & Liu (2002 ▶), Qi *et al.* (2005 ▶). For properties and applications of dialdehydes, see: Ma & Liu (2003*a* ▶,*b* ▶); Ma & Cao (2005 ▶); Ragunathan & Bharadwaj (1992 ▶); Ray & Bharadwaj (2006 ▶). For standard bond lengths, see: Allen *et al.* (1987 ▶).
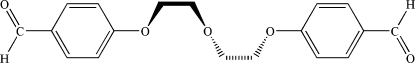


## Experimental

### 

#### Crystal data

C_18_H_18_O_5_
*M* *_r_* = 314.32
Monoclinic, 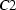

*a* = 15.309 (2) Å
*b* = 4.5653 (6) Å
*c* = 11.8332 (15) Å
β = 113.253 (7)°
*V* = 759.85 (17) Å^3^
*Z* = 2
Mo *K*α radiation
μ = 0.10 mm^−1^
*T* = 298 K
0.32 × 0.26 × 0.23 mm

#### Data collection

Bruekr SMART CCD area-detector diffractometer
Absorption correction: multi-scan (*SADABS*; Sheldrick, 1996 ▶) *T*_min_ = 0.969, *T*_max_ = 0.977
8239 measured reflections
1566 independent reflections
1374 reflections with *I* > 2σ(*I*)
*R*_int_ = 0.026

#### Refinement

*R*[*F* ^2^ > 2σ(*F* ^2^)] = 0.036
*wR*(*F* ^2^) = 0.101
*S* = 1.07
1566 reflections
105 parameters
1 restraint
H-atom parameters constrained
Δρ_max_ = 0.35 e Å^−3^
Δρ_min_ = −0.19 e Å^−3^

### 

Data collection: *SMART* (Bruker, 2001 ▶); cell refinement: *SAINT* (Bruker, 2002 ▶); data reduction: *SAINT*; program(s) used to solve structure: *SHELXS97* (Sheldrick, 2008 ▶); program(s) used to refine structure: *SHELXL97* (Sheldrick, 2008 ▶); molecular graphics: *SHELXTL* (Sheldrick, 2008 ▶); software used to prepare material for publication: *SHELXL97*.

## Supplementary Material

Crystal structure: contains datablocks I, global. DOI: 10.1107/S1600536811018150/zl2368sup1.cif

Structure factors: contains datablocks I. DOI: 10.1107/S1600536811018150/zl2368Isup2.hkl

Supplementary material file. DOI: 10.1107/S1600536811018150/zl2368Isup3.cml

Additional supplementary materials:  crystallographic information; 3D view; checkCIF report

## Figures and Tables

**Table 1:** Hydrogen-bond geometry (Å, °) *Cg* is the centroid of the C2–C7 ring.

*D*—H⋯*A*	*D*—H	H⋯*A*	*D*⋯*A*	*D*—H⋯*A*
C9—H9*B*⋯O1^i^	0.97	2.58	3.3772 (18)	139
C4—H4*A*⋯O2^ii^	0.93	2.59	3.3434 (16)	139
C8—H8*B*⋯*Cg*^iii^	0.97	3.14	3.7172 (14)	129

Symmetry codes: (i) 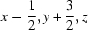; (ii) 
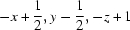
; (iii) 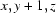.

**Fig. 1.**
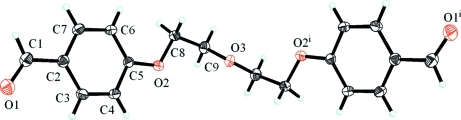
The molecular structure of the title compound with the atom numbering scheme. Displacement ellipsoids are drawn at the 50% probability level. H atoms are presented as small spheres of arbitrary radius. [symmetry code: (i) -x, y, 1-z]

**Fig. 2.**
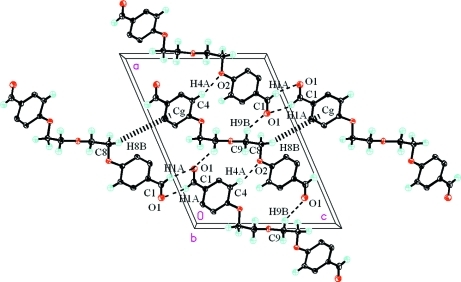
A view of the crystal packing along the b axis. Some H atoms were omitted for clarity. The thin dashed lines are used to show the hydrogen bonds and the intermolecular CH-π interactions of the two carbonyl groups. The thick dashed line is used to show the intermolecular CH-π interactions of –CH2-(C8) and the neighboring aryl groups, from their H atoms to the centroids of the rings of the aryl groups.

**Fig. 3.**
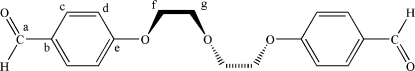
An additional scheme with the numbering scheme used for the NMR spectra.

## supplementary crystallographic information

### Comment

There has been, in recent years, a considerable interest in the study of aldehydes (Aravindan *et al.*, 2003; Han & Zhen 2005; Qi *et al.*, 2005), since these compounds are commodity chemicals used as intermediates in the manufacture of acids or alcohols, and to produce many important industrial products. Aldehydes are also used as important precusors in the synthesis of macrocyclic or/and macrobicyclic compounds by [1 + 1], [2 + 2] or [2 + 3] condensation with polyamines (Ma & Liu, 2003*a*; Ma & Liu, 2003*b*; Ma & Cao, 2005; Ragunathan & Bharadwaj, 1992; Ray & Bharadwaj, 2006). Hence, the current work aims to prepare dialdehydes and trialdehydes, to investigate their condensation behaviors with oligo-amines and synthesize macrocyclic and/or macrobicyclic compounds. Herein, we report a new dialdehyde which was obtained by reaction of 4-hydroxybenzaldehyde with bis(2,2'-dichloroethyl)ether in DMF and its structure was confirmed by elemental analysis, IR, NMR spectra and X-ray crystal analysis.

The structure consists of a neutral molecular unit (Fig. 1). A crystallographic twofold rotation axis passes through the central O atom of the aliphatic chain and there is thus one half molecule in the asymmetric unit. All bond lengths and angles are within normal ranges (Allen *et al.*, 1987). The two aromatic rings are approximately perpendicular to each other with the dihedral angle being 78.31 (2) °. The aryl, carbonyl and the O—CH_2_—CH_2_ groups of one half molecule are coplanar to form one plane with an r.m.s. deviation of 0.030 Å. The planes of the two halves of the molecule are bent at the C—O—C atoms with angles of 109.2 (1) [for C8—C9—O3], 110.3 (2) [for C9—O3—C9^i^], and 109.2 (1) ° [for O3—C9^i^—C8^i^, symmetry code: (i) -*x*, *y*, 1-*z*], respectively, forming a "w" structure for the whole molecule (see Fig 2). Two weak hydrogen bonds are present in the structure between two hydrogen atoms and two oxygen atoms of neighboring molecules: H9B at C9 and O1^ii^ [symmetry code: (ii) *x*-1/2, *y*+3/2, *z*], and H4A at C4 and O2^iii^,[symmetry code: (iii) -*x*+1/2, *y*-1/2, -*z*+1], respectively (Table 1). The molecules display two kinds of intermolecular CH-π interactions. One is between the aldehyde C—H group of C1 and the π system of the same aldehyde in a neighboring molecule [H1A···C1^iv^ = 2.827 Å, C1···C1^iv^ = 3.454 (2) Å, symmetry code: (iv) -*x*+1/2, *y*+1/2, -*z*]. The other is between the –CH_2_- group of C8 and a neighboring aryl group [H8B···Cg^v^ = 3.139 Å, Cg is the centroid of the six membered ring of C2-C7, symmetry code: (v) *x*, *y*+1, *z*].

### Experimental

All synthetic processes were undertaken under dinitrogen gas. The title compound was obtained by the reaction of 4-hydroxybenzaldehyde with bis(2,2'-dichloroethyl)ether in *N*,*N*'-dimethylformamide (DMF). In a 100 cm^3^ flask fitted with a funnel, 4-hydroxy-benzaldehyde (6.1 g, 50 m*M*) and potassium carbonate were mixed in 50 cm^3^ of DMF. To this solution was added dropwise a stoichiometric quantity of bis(2,2'-dichloroethyl)ether (3.6 g, 25 m*M*) dissolved in 20 cm^3^ of DMF for a period of an hour with stirring. The mixture was then stirred for 24 h at 353 K. The solution was concentrated under reduced pressure and the white solid formed by adding a large quantity of water (200 cm^3^) was filtered off and recrystallized from ethanol and decolored with activated carbon. A colorless solid was obtained (Yield 81%, m.p: 363–365 K). Slow evaporation of a solution of the title compound in ethanol and dichloromethane (1:1) led to the formation of colorless crystals, which were suitable for X-ray characterization. Anal. Calcd. for [C_18_H_18_O_5_] (%): C, 68.78; H, 5.77; found: C, 68.49; H, 5.93; IR (KBr), (cm^-1^): 3100 (C—H of aryl), 2730 (C—H of –CHO), 1695 (C=O), 1500, 1490 (C=C of aryl), 1150, 1176, 1260 (CH_2_—O—CH_2_), 985, 860–705 (Ar—H). ^1^H NMR (CDCl_3_): 9.87 (s, 2H, CHO), 7.81 (d, 4H, *J* = 8.4 Hz, aryl, c), 7.02 (d, 4H, *J* = 8.8 Hz, aryl, d), 4.22 (d, 4H, *J* = 4.8 Hz, O—CH_2_CH_2_, f), 3.97 (d, 4H, *J* = 4.8 Hz, –CH_2_, g). ^13^C NMR: 191.09 (–CHO,a), 164.04 (aryl, b), 132.29 (aryl, c), 130.51 (aryl, d), 115.20 (aryl, e), 70.10 (–CH_2_CH_2_, f), 68.08 (–CH_2_CH_2_, g) (see figure 3 for the NMR atom number assignment).

### Refinement

All H atoms were positioned geometrically and refined using a riding model with C—H = 0.93 - 0.97 Å and with *U*_iso_(H) = 1.2 times *U*_eq_(C). In the absence of significant anomalous scatterers 1085 Friedel pairs were merged prior to refinement.

### Crystal data

**Table:**

C_18_H_18_O_5_	*F*(000) = 332
*M**_r_* = 314.32	*D*_x_ = 1.374 Mg m^−^^3^
Monoclinic, *C*2	Mo *K*α radiation, λ = 0.71073 Å
Hall symbol: C2y	Cell parameters from 8239 reflections
*a* = 15.309 (2) Å	θ = 2.8–33.2°
*b* = 4.5653 (6) Å	µ = 0.10 mm^−^^1^
*c* = 11.8332 (15) Å	*T* = 298 K
β = 113.253 (7)°	Prism, colorless
*V* = 759.85 (17) Å^3^	0.32 × 0.26 × 0.23 mm
*Z* = 2	

### Data collection

**Table:**

Bruekr SMART CCD area-detector diffractometer	1566 independent reflections
Radiation source: fine-focus sealed tube	1374 reflections with *I* > 2σ(*I*)
Graphite Monochromator	*R*_int_ = 0.026
Detector resolution: 0 pixels mm^-1^	θ_max_ = 33.2°, θ_min_ = 2.8°
φ and ω scans	*h* = −22→22
Absorption correction: multi-scan (*SADABS*; Sheldrick, 1996)	*k* = −6→6
*T*_min_ = 0.969, *T*_max_ = 0.977	*l* = −18→17
8239 measured reflections	

### Refinement

**Table:**

Refinement on *F*^2^	Primary atom site location: structure-invariant direct methods
Least-squares matrix: full	Secondary atom site location: difference Fourier map
*R*[*F*^2^ > 2σ(*F*^2^)] = 0.036	Hydrogen site location: inferred from neighbouring sites
*wR*(*F*^2^) = 0.101	H-atom parameters constrained
*S* = 1.07	*w* = 1/[σ^2^(*F*_o_^2^) + (0.0634*P*)^2^ + 0.1116*P*] where *P* = (*F*_o_^2^ + 2*F*_c_^2^)/3
1566 reflections	(Δ/σ)_max_ < 0.001
105 parameters	Δρ_max_ = 0.35 e Å^−^^3^
1 restraint	Δρ_min_ = −0.19 e Å^−^^3^

### Special details

**Table:**

Geometry. All e.s.d.'s (except the e.s.d. in the dihedral angle between two l.s. planes) are estimated using the full covariance matrix. The cell e.s.d.'s are taken into account individually in the estimation of e.s.d.'s in distances, angles and torsion angles; correlations between e.s.d.'s in cell parameters are only used when they are defined by crystal symmetry. An approximate (isotropic) treatment of cell e.s.d.'s is used for estimating e.s.d.'s involving l.s. planes.
Refinement. Refinement of *F*^2^ against ALL reflections. The weighted *R*-factor *wR* and goodness of fit *S* are based on *F*^2^, conventional *R*-factors *R* are based on *F*, with *F* set to zero for negative *F*^2^. The threshold expression of *F*^2^ > σ(*F*^2^) is used only for calculating *R*-factors(gt) *etc*. and is not relevant to the choice of reflections for refinement. *R*-factors based on *F*^2^ are statistically about twice as large as those based on *F*, and *R*- factors based on ALL data will be even larger.

### Fractional atomic coordinates and isotropic or equivalent isotropic displacement parameters (Å^2^)

**Table:**

	*x*	*y*	*z*	*U*_iso_*/*U*_eq_	
O2	0.11077 (6)	0.9413 (2)	0.36300 (8)	0.0224 (2)	
O3	0.0000	1.0762 (3)	0.5000	0.0188 (3)	
C8	0.01664 (9)	1.0616 (3)	0.30796 (12)	0.0202 (2)	
H8A	−0.0303	0.9059	0.2837	0.024*	
H8B	0.0095	1.1751	0.2356	0.024*	
C2	0.21612 (9)	0.3700 (3)	0.17623 (12)	0.0196 (3)	
C5	0.14083 (9)	0.7593 (3)	0.29553 (11)	0.0180 (2)	
C4	0.23027 (9)	0.6328 (3)	0.35916 (12)	0.0214 (3)	
H4A	0.2646	0.6791	0.4414	0.026*	
C7	0.12779 (9)	0.4969 (3)	0.11397 (12)	0.0219 (3)	
H7A	0.0936	0.4503	0.0317	0.026*	
C1	0.25348 (10)	0.1644 (3)	0.11128 (13)	0.0246 (3)	
H1A	0.2136	0.1144	0.0313	0.029*	
C6	0.08902 (9)	0.6923 (3)	0.17174 (11)	0.0207 (3)	
H6A	0.0298	0.7767	0.1288	0.025*	
O1	0.33176 (7)	0.0537 (3)	0.15259 (10)	0.0313 (3)	
C9	0.00335 (10)	1.2542 (3)	0.40302 (12)	0.0215 (3)	
H9A	0.0556	1.3920	0.4353	0.026*	
H9B	−0.0553	1.3645	0.3659	0.026*	
C3	0.26761 (9)	0.4399 (3)	0.30040 (12)	0.0211 (3)	
H3A	0.3270	0.3561	0.3431	0.025*	

### Atomic displacement parameters (Å^2^)

**Table:**

	*U*^11^	*U*^22^	*U*^33^	*U*^12^	*U*^13^	*U*^23^
O2	0.0160 (4)	0.0308 (5)	0.0187 (4)	0.0001 (4)	0.0050 (3)	−0.0039 (4)
O3	0.0237 (6)	0.0179 (6)	0.0170 (6)	0.000	0.0105 (5)	0.000
C8	0.0179 (5)	0.0244 (6)	0.0173 (5)	0.0009 (5)	0.0060 (4)	0.0024 (5)
C2	0.0208 (6)	0.0204 (6)	0.0186 (6)	−0.0009 (5)	0.0088 (5)	0.0009 (5)
C5	0.0167 (5)	0.0210 (6)	0.0168 (5)	−0.0032 (5)	0.0073 (4)	0.0003 (5)
C4	0.0173 (5)	0.0288 (7)	0.0159 (5)	−0.0016 (5)	0.0040 (4)	−0.0003 (5)
C7	0.0221 (6)	0.0264 (7)	0.0152 (6)	−0.0008 (5)	0.0051 (5)	−0.0001 (5)
C1	0.0291 (6)	0.0239 (6)	0.0212 (6)	0.0019 (5)	0.0105 (5)	0.0006 (5)
C6	0.0169 (5)	0.0264 (7)	0.0158 (5)	0.0005 (5)	0.0033 (4)	−0.0002 (5)
O1	0.0307 (5)	0.0330 (6)	0.0315 (6)	0.0073 (5)	0.0136 (4)	−0.0004 (5)
C9	0.0250 (6)	0.0191 (6)	0.0222 (6)	0.0013 (5)	0.0112 (5)	0.0029 (5)
C3	0.0165 (5)	0.0257 (6)	0.0195 (6)	0.0003 (5)	0.0055 (4)	0.0025 (5)

### Geometric parameters (Å, °)

**Table:**

O2—C5	1.3528 (16)	C5—C4	1.4004 (18)
O2—C8	1.4357 (15)	C4—C3	1.378 (2)
O3—C9^i^	1.4235 (16)	C4—H4A	0.9300
O3—C9	1.4235 (16)	C7—C6	1.3915 (19)
C8—C9	1.504 (2)	C7—H7A	0.9300
C8—H8A	0.9700	C1—O1	1.2114 (17)
C8—H8B	0.9700	C1—H1A	0.9300
C2—C7	1.3857 (18)	C6—H6A	0.9300
C2—C3	1.4027 (18)	C9—H9A	0.9700
C2—C1	1.465 (2)	C9—H9B	0.9700
C5—C6	1.3967 (17)	C3—H3A	0.9300
			
C5—O2—C8	118.79 (10)	C2—C7—H7A	119.2
C9^i^—O3—C9	110.38 (15)	C6—C7—H7A	119.2
O2—C8—C9	107.00 (11)	O1—C1—C2	125.76 (13)
O2—C8—H8A	110.3	O1—C1—H1A	117.1
C9—C8—H8A	110.3	C2—C1—H1A	117.1
O2—C8—H8B	110.3	C7—C6—C5	118.66 (12)
C9—C8—H8B	110.3	C7—C6—H6A	120.7
H8A—C8—H8B	108.6	C5—C6—H6A	120.7
C7—C2—C3	119.25 (12)	O3—C9—C8	109.13 (12)
C7—C2—C1	119.35 (12)	O3—C9—H9A	109.9
C3—C2—C1	121.40 (12)	C8—C9—H9A	109.9
O2—C5—C6	124.66 (12)	O3—C9—H9B	109.9
O2—C5—C4	115.10 (11)	C8—C9—H9B	109.9
C6—C5—C4	120.24 (12)	H9A—C9—H9B	108.3
C3—C4—C5	120.29 (12)	C4—C3—C2	120.03 (12)
C3—C4—H4A	119.9	C4—C3—H3A	120.0
C5—C4—H4A	119.9	C2—C3—H3A	120.0
C2—C7—C6	121.53 (12)		
			
C5—O2—C8—C9	−179.71 (11)	C2—C7—C6—C5	−0.3 (2)
C8—O2—C5—C6	4.4 (2)	O2—C5—C6—C7	−178.83 (13)
C8—O2—C5—C4	−174.93 (12)	C4—C5—C6—C7	0.5 (2)
O2—C5—C4—C3	178.97 (12)	C9^i^—O3—C9—C8	174.42 (13)
C6—C5—C4—C3	−0.4 (2)	O2—C8—C9—O3	−68.53 (13)
C3—C2—C7—C6	0.1 (2)	C5—C4—C3—C2	0.2 (2)
C1—C2—C7—C6	179.68 (12)	C7—C2—C3—C4	0.0 (2)
C7—C2—C1—O1	174.98 (15)	C1—C2—C3—C4	−179.59 (13)
C3—C2—C1—O1	−5.4 (2)		

Symmetry codes: (i) −*x*, *y*, −*z*+1.

### Hydrogen-bond geometry (Å, °)

**Table:**

Cg is the centroid of the C2–C7 ring.

**Table:**

*D*—H···*A*	*D*—H	H···*A*	*D*···*A*	*D*—H···*A*
C9—H9B···O1^ii^	0.97	2.58	3.3772 (18)	139
C4—H4A···O2^iii^	0.93	2.59	3.3434 (16)	139
C8—H8B···Cg^iv^	0.97	3.14	3.7172 (14)	129

Symmetry codes: (ii) *x*−1/2, *y*+3/2, *z*; (iii) −*x*+1/2, *y*−1/2, −*z*+1; (iv) *x*, *y*+1, *z*.
